# Cytomegalovirus in the Neonate: Immune Correlates of Infection and Protection

**DOI:** 10.1155/2013/501801

**Published:** 2013-08-19

**Authors:** Mark R. Schleiss

**Affiliations:** Department of Pediatrics, University of Minnesota Medical School, Center for Infectious Diseases and Microbiology Translational Research, 2001 6th Street SE, Minneapolis, MN 55455, USA

## Abstract

Fetal and neonatal infections caused by human cytomegalovirus (CMV) are important causes of morbidity and occasional mortality. Development of a vaccine against congenital CMV infection is a major public health priority. Vaccine design is currently focused on strategies that aim to elicit neutralizing antibody and T-cell responses, toward the goal of preventing primary or recurrent infection in women of child-bearing age. However, there has been relatively little attention given to understanding the mechanisms of immune protection against acquisition of CMV infection in the fetus and newborn and how this information might be exploited for vaccine design. There has similarly been an insufficient study of what deficits in the immune response to CMV, both for mother and fetus, may increase susceptibility to congenital infection and disease. Protection of the fetus against vertical transmission can likely be achieved by protection of the placenta, which has its own unique immunological milieu, further complicating the analysis of the correlates of protective immunity. In this review, the current state of knowledge about immune effectors of protection against CMV in the maternal, placental, and fetal compartments is reviewed. A better understanding of immune responses that prevent and/or predispose to infection will help in the development of novel vaccine strategies.

## 1. Introduction

Human cytomegalovirus (CMV) is the most common cause of congenital viral infection in the developed world, occurring in 0.5–2% of pregnancies in the United States and Europe [1, 2]. Congenital infections can cause severe sequelae among neonates including sensorineural hearing loss, cerebral palsy, microcephaly, cognitive impairments, and mental retardation [3–5]. During maternal primary infection, and to a lesser extent during recurrent infection, CMV can translocate the placental barrier and can cause infection of the developing fetus [6, 7]. Infection acquired *in utero* may have no clinical manifestations, or may manifest with hepatosplenomegaly, thrombocytopenia, cholestatic hepatitis, petechiae and purpura, central nervous system pathologies (including retinitis), viremia, and pneumonia [8]. In addition to being at risk for severe, occasionally life-threatening end-organ disease [9], infants with symptoms at birth also have an increased risk for long-term neurodevelopmental sequelae, including sensorineural hearing loss (SNHL). The long-term neurodevelopmental prognosis of a congenitally infected infant depends upon a number of factors, including the maternal immune status prior to the onset of pregnancy, whether or not she is reinfected with a new strain of CMV during pregnancy, and the timing of acquisition of fetal infection [10–12]. 

In addition to the impact of CMV infections acquired *in utero*, postnatal acquisition of CMV can also cause significant morbidity and occasional mortality. Disease is typically not observed in term infants, but can be a substantial problem for low birth weight premature infants [13, 14]. Because of the virtual elimination of transfusion-associated CMV heralded by the advent of leukofiltration of blood products [15], essentially all CMV infections in premature infants are acquired from maternal breast milk [16–18]. As is the case for congenital CMV infections, many breast milk-acquired infections in premature infants are asymptomatic, but a substantial percentage can produce severe, occasionally life-threatening disease, which can manifest as viremia, neutropenia, thrombocytopenia, hepatitis, pneumonia, enteritis, and a sepsis-like syndrome [14, 19]. It remains unclear whether such postnatal acquisition of infection poses any long-term risk for adverse neurodevelopmental outcomes [20–23].

**Figure 1:**
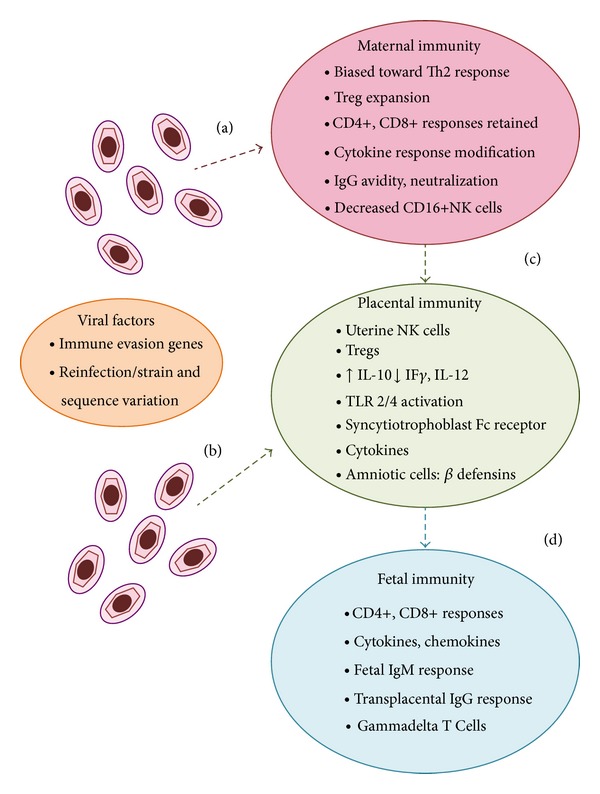
Schematic representation of pathways of CMV to the fetus and immune responses potentially important in transmission and prevention. The figure emphasizes the immunological milieu of pregnancy and some of the known immune adaptations associated with pregnancy. Left side of figure, CMV is known both to encode a plethora of immune evasion genes that subvert immune clearance of infection and to demonstrate substantial strain variation that can promote reinfection of already-immune hosts. Virus is believed to reach the placenta via the maternal compartment (a) or ascending infection via local extension in the reproductive tract (b). Although maternal antibody, CD4+, and CD8+ responses are generally intact in pregnancy, there are alterations in Th1/Th2 cytokine balance; alterations in NK cell subpopulations; increased Tregs; and modified cytokine responses. The uterine microenvironment in pregnancy may also play a role in direct local extension of CMV following virus exposure or reactivation (b), driven in part by increased localized IL-10 expression. Irrespective of the route of infection, the immunological profile of the placenta may either facilitate CMV transmission or inhibit it. Factors that may promote transmission include the less efficient killing potential of uNK cells; decreases in cytokines such as IL-12 and IF-*γ*; and the potential translocation of CMV particles across the syncytiotrophoblast if low avidity IgG is present. Factors that inhibit transmission include chemokines and *β*-defensins and, if present, high avidity neutralizing antibody, which may render virus noninfectious. Once virus enters the fetal compartment, the impaired capacity of fetal CD4+ to proliferate in response to CMV may impair immune control. The presence of transplacentally acquired IgG is believed to ameliorate the severity of disease. There is evidence that CD8+ cells, chemokines, and gammadelta T cells contribute to antiviral immunity in the fetal immune environment.

Although the risks of CMV infection to the developing fetus and neonate are well recognized, the factors that dictate whether or not an infant has an asymptomatic infection or manifests with severe disease are not clear. Immune protection against congenital CMV infection is complex and requires consideration of immune responses in the mother, the fetus, and the placenta (Figure 1). Consideration must also be given to the burgeoning list of virally encoded immune modulation and immune evasion genes, which almost certainly exert a clinically relevant impact on the maternal and fetal immune responses to infection [24]. Another important issue is that of the problem of viral strain variation and attendant reinfection, in light of the emerging recognition that maternal antiviral immunity to one strain of CMV may not protect against acquisition of, and subsequent fetal transmission with, a new strain [7, 25, 26]. Development of a vaccine against congenital CMV infection is a major public health priority [27], but most vaccine approaches to date have focused on what is probably an overly simplistic approach: namely, the prevention of primary infection in young women of child-bearing age. Although clearly infection of the fetus cannot occur if maternal infection is prevented by a successful vaccine [28, 29], current vaccine approaches have, by focusing almost exclusively on the prevention of maternal infection, failed to take into consideration the incompletely understood but critically important fetal and newborn immune responses to CMV that may play key roles in preventing CMV end-organ disease and sequelae. Understanding the immune response of the infected fetus may facilitate identification of correlates of protective immunity in the infant. Moreover, it is important to note that many of the CMV vaccines currently in clinical trials are focused on inducing immune responses known to be important in controlling CMV disease in solid organ and hematopoietic stem cell transplant patients, but these effectors of protection in transplant patients may or may not be relevant to the problem of prevention of maternal and fetal CMV infections [30]. 

Maternal-placental-fetal transmission of CMV occurs against the backdrop of the altered cytokine state of pregnancy, in which there is a functional immune suppression mediated via a shift from a Th1 response to a Th2 bias [31–33]. The altered immune state in pregnancy is likely to be highly relevant to the problem of sustaining vaccine-mediated protection against infection and transmission. Issues relevant to the study of the immunology of maternal-fetal CMV transmission are germane to the question of the key clinical and immunologic endpoints of vaccine trials, and may help define the most suitable patient population for ultimate administration of a licensed CMV vaccine against congenital CMV infection. A universal vaccine meant to provide a broad blanket of herd immunity on a population of young children may have different requirements than a vaccine selectively targeting young women of reproductive age. Acceptable attributes of a CMV vaccine in the latter scenario may include not only protection against transmission of infection, but also mediation of protection against CMV disease, even if transmission occurs. Congenital infection commonly occurs in the absence of symptoms or signs of illness, and asymptomatic infants generally have an excellent prognosis for normal neurodevelopment. Thus, better insight into those aspects of the immune response that may contribute to protection against disease in the asymptomatically infected infant might help guide future immunization approaches. This review summarizes recent observations gleaned from the study of the immune response to CMV, focusing on the pregnant woman and developing fetus. The potential application of these studies to immunotherapeutic interventions for prevention of congenital CMV infection and long-term disability is also discussed.

## 2. Innate Immunity

Innate immunity likely plays a crucial role in preventing acquisition of congenital and perinatal CMV infections; conversely, the failure of innate immunity, either due to host genetic factors, the immune tolerance state of pregnancy, and/or viral immune evasion, may contribute to an increased risk of acquisition of infection. Components of the innate immune response in the setting of congenital and perinatal infection include natural killer (NK) cells; toll-like receptors (TLRs); and cytokines. Available information about the role of each of these components in congenital and perinatal CMV infection and transmission is considered below.

### 2.1. NK Cells

NK cells are important effectors of innate immunity involved in control of viral infection. Human NK cells are typically characterized as CD3−CD56+ lymphocytes that make up about 15% of peripheral blood lymphocytes. They are further subdivided into CD56^bright^ cells (lacking the expression of CD16 and the killer immunoglobulin-like receptor, KIR) and CD56^dim⁡^ cells, which express CD16 and KIR [34]. NK cells target virally infected cells through perforin and granzyme-mediated cell killing, Fas-ligand initiated apoptosis, and antibody dependent cellular cytotoxicity (ADCC). They also elaborate cytokines and modulate adaptive immune responses via interactions with plasmacytoid dendritic cells [35, 36]. CMV encodes a number of genes that interfere with the NK cell response. Some of these viral genes encode proteins that alter expression of NK cell receptor ligands, resulting in perturbation of function of the activating receptor, NKG2D; proteins that are homologs of MHC class I that bind the NK cell inhibitory receptor, LIR-1, with a higher affinity than host MHC class I; and proteins that decrease the expression of CD155, a ligand for NK cell activating receptors [24].

Modulation of the distribution of subtypes of NK cells during pregnancy may have an impact on the risk for acquisition of CMV infection by the placental-fetal unit. During pregnancy, the uterus contains cells known as “uterine NK cells (uNK)” or “decidual NK cells (dNK)”. Although these differ from peripheral NK cells, they are in the CD56^bright^ subset of NK cells, and hence have lower cytotoxic ability, similar to peripheral CD56^bright^ NK cells [37]. This is one of many different manifestations of the “immune tolerance” state required during pregnancy to prevent rejection of the fetal allograft [38]. During the first trimester of pregnancy, uNK cells are the major population of maternal immune cells, accounting for ~70% immune cells in the decidua, with macrophages, T cells (CD8+, CD4+, and *γδ*T cells), and dendritic cells accounting for 20, 10, and 2%, respectively [39–41]. These uNK cells may compensate for their relative lack of cytotoxic potential by elaboration of antiviral cytokines, particularly interferon gamma, in the uterine microenvironment [42]. It has been reasonably speculated that the lack of an effector phenotype for the uNK cells may contribute to an increased risk of intrauterine CMV transmission [43]. However, it has also been noted that freshly isolated uNK cells can acquire major functional and phenotypic changes and can become cytotoxic effectors following exposure to CMV-infected autologous decidual fibroblasts. NKG2D+ and CD94/NKG2C+ or 2E+ activating receptors are involved in the acquisition of cytotoxic function, and these cells in an *ex vivo* model of CMV-infected trophoblast colocalize with CMV-infected cells [44]. Hence, the cytotoxic potential of these cells following exposure to virus may be important in prevention of CMV transmission in early pregnancy [45].

In addition to the role NK cells play in the placental environment, a suboptimal or deficient NK cell response may play a role in modulating the clinical manifestations and severity of congenital CMV infection. A child with NK cell deficiency was noted to have severe herpesvirus infections, including CMV, although her CMV infection did not appear to be acquired in the perinatal period [46]. A deficiency in NK cell cytotoxic response to herpes simplex virus (HSV)-infected cells was proposed to be a predisposing factor influencing the severity of neonatal HSV infection [47]; whether such mechanisms are relevant for perinatally acquired CMV infection remains to be evaluated. A recent study demonstrated that increased proportions of NK cells expressing the activating killer lectin-like receptor, NKG2C+, were more frequently detected in children with congenital CMV infection. Strikingly, this immunophenotype was more common in symptomatic cases of congenital infection [48], suggesting this as an important correlate of disease outcome. Expansion of NKG2C+ cells also appeared more marked in children with postnatal infection (presumed to be acquired by breastfeeding) than in the group of infants with congenital asymptomatic infection. Based on analogy with studies performed in immune suppressed patients, the authors speculated that the magnitude of the NKG2C+ expansion might be inversely related to the effectiveness of the T-cell response to CMV infection; in other words, that NKG2C+ expansion might reflect inadequate T-cell immunity. Immunophenotyping of NK responses, therefore, might prove useful in assessing prognosis, or identifying infants that would be candidates for immunotherapies. Whether the expansion of NKG2C+ NK cells observed in the setting of symptomatic congenital or perinatal infection contributes to the immunopathogenesis, or conversely the long-term disease control of CMV infection, will require further study.

### 2.2. Phagocytic Cells

There is relatively little information about the role of phagocytic cells (neutrophils, macrophages) in protection against congenital infection or, in the setting of aberrant function, increased susceptibility to congenital infection. That neutrophils may be important in the first line of defense against vertical transmission of infection is suggested by pathologic studies of CMV-infected placentas demonstrating neutrophilic infiltrates in fetal blood vessels in the villus core [49]. In these studies, placentas with high levels of viral DNA were associated with neutrophilic infiltrations, whereas macrophages and dendritic cells were associated with low levels of DNA; hence, a response biased toward a phagocytic cellular response may be associated with less robust control of infection. Notably, congenital CMV infection does not appear to be associated with heritable abnormalities in neutrophilic oxidative burst, as seen in chronic granulomatous disease [50]. A case of congenital CMV was recently described in a patient with leukocyte adhesion defect type 1 [51], although this infant also had a natural killer cell deficiency, making it unclear to what extent the neutrophil defect contributed to the increased risk of CMV infection. In another case of fulminant congenital CMV, delayed fetal neutrophil differentiation was implicated as a possible contributing factor to the fatal outcome [52].

No cases of congenital CMV infection appear to have been correlated with aberrant macrophage function. However, macrophages do appear to play an as-yet incompletely defined role in modulating vertical transmission of CMV. In CMV infected placentas, marked hyperplasia of fetal-derived placental macrophages, termed Hofbauer cells, has been observed [53]. A serum proteomic comparison of infants with congenital CMV infection and controls demonstrated upregulation of a macrophage-derived chemokine in those infants with CMV infection [54]. It has been suggested that macrophages may potentiate CMV infection and spread in syncytiotrophoblasts. This is based on a cell coculture model system in which the presence of macrophages enhanced activation of CMV in syncytiotrophobast and promoted transmission of virus from cell-to-cell, an effect which was mediated by IL-8 and TGF-*β* [55]. Macrophages are also themselves targets of CMV infection. Viral antigen was noted in macrophages in a study of term CMV-infected placentas [49]. The ability of CMV to infect macrophages was also demonstrated in a recently described decidual organ culture model [56].

### 2.3. Toll-Like Receptors (TLRs)

There are ten toll-like receptors (TLRs) described in humans [57]. TLRs function to sense microbial pathogens through interactions of pathogen-associated molecular patterns (PAMPs) through their cognate pattern recognition receptors (PRRs), in the process signaling through MyD88-dependent and TRIF-dependent pathways, which in turn upregulates cytokine production [58]. There is evidence that interactions between some members of the TLR family with CMV influence both the immune response to, as well as the outcome of, infection.

TLR2, although typically considered in the context of PAMPs consisting of bacterial polysaccharides, is also a PRR for the CMV envelope glycoproteins, glycoprotein B (gB), and glycoprotein H (gH). Notably, a polymorphism in the TLR2 gene was shown to be associated with increased CMV replication and an increased risk of CMV disease in liver transplant recipients [59, 60]. No data exist, however, on whether this TLR2 polymorphism impacts the phenotype of fetal or perinatal CMV disease. CMV-mediated TLR2 signaling was noted to lead to an inflammatory response in a cell culture model utilizing syncytiotrophoblasts, suggesting that this pathway could have some influence on the manifestations of placental-fetal infection *in vivo* [61, 62]. This signaling occurred in the absence of viral replication, indicating that structural component(s) of the virion may be responsible. These structural components appear to be gB and gH, which have been shown to induce inflammatory cytokine secretion in response to CMV exposure, independent of viral replication [63, 64]. This signaling appears to be mediated by a TLR heterodimer on the infected cell surface, consisting of TLR2 and TLR1 [63]. 

TLR3 interactions with CMV are related to the binding of double stranded RNA molecules produced during CMV replication [57]. The interrelationship between polymorphisms in TLR3 and susceptibility to herpesvirus disease was demonstrated in studies linking HSV encephalitis to a specific TLR3 polymorphism associated with diminished inflammatory cytokine production following stimulation with an agonist [65, 66]. This diminished cytokine responsiveness in turn correlated with an increased severity of herpes simplex encephalitis. Recently, an increased susceptibility to CMV infection was proposed for individuals with the L412F variant of TLR3 [67]. Peripheral blood mononuclear cells from these patients, who are known to have an increased risk for fungal infections and autoimmunity, were assayed for secretion of cytokines in response to TLR3 ligands and to CMV. Reduced IFN*γ* and TNF*α* secretion were demonstrated when the L412F polymorphism was present. It was inferred that this TLR3 polymorphism conferred an increased risk of CMV disease [67]. On the other hand, no role for TLR3 signaling could be demonstrated during the early immune response of human monocyte-derived dendritic cells after infection with a wild-type isolate of CMV, strain TB40E. This study therefore failed to support a role for CMV-TLR3 interactions in the immunopathogenesis of infection [68].

TLR7 polymorphisms have been suggested to play a role in dictating the magnitude of the immune response to CMV glycoproteins. In a study genotyping 142 women who had been previously vaccinated with three doses of adjuvanted CMV gB vaccine, it was observed that homozygous carriers of single nucleotide polymorphisms in TLR7 demonstrated a higher vaccination-induced antibody response to gB than did heterozygotes or homozygotes for this allele [69]. Whether or not TLR7 polymorphisms impact the immune response to gB in the context of natural CMV infection, or transmission of CMV to the fetus, remains to be evaluated.

### 2.4. Cytokines, Chemokines, and Defensins

Many cytokines appear to be important in immune control of CMV infection, although defining cytokine(s) that may correlate either with protection or increased susceptibility to infection in the context of congenital or perinatal CMV infection has been difficult in studies reported to date. One difficulty in interpreting studies correlating altered cytokine profiles with an increased CMV infection risk is that such associations may not reflect isolated cytokine perturbations *per se*, but rather modulation of upstream events that trigger (or diminish) the elaboration of cytokines. These include the TLR polymorphisms already discussed, such as the TLR3 polymorphisms that are associated with decreased proinflammatory cytokine production [67]. Physiologic alterations in cytokine production during pregnancy may contribute to an increased risk of some viral infections. Normal pregnancy is also associated with increased production of IL-10 [70], an anti-inflammatory cytokine, and increased production of this cytokine during pregnancy has been proposed to increase susceptibility to fetal CMV infection [43]. Other cytokines proposed to be important in the context of perinatal viral transmission include TNF-*α*, IL-12, IL-17, IL-18, IL-23, and IL-1*β* [43, 71], although there has been no clear correlation between hereditary deficiencies in any of these cytokines with an increased risk of congenital infection. Similarly, therapeutic monoclonal antibodies targeting TNF-*α* have not been associated with an increased risk of congenital CMV. However, it has been pointed out that the use of TNF-*α* blocking agents during pregnancy may, by blocking the activity of the TNF superfamily members lymphotoxin-*α* and -*β*, negatively impact the development and organization of secondary lymphoid tissues [72]. A recent analysis of the safety of these agents during pregnancy and lactation suggested an increased risk of infection in infants exposed to these monoclonal antibodies [73], although CMV was not specifically mentioned.

An important consideration in the analysis of the cytokine profile of the congenitally CMV-infected infant is the fact that infant immunocytes produce smaller amounts of cytokines than do comparable adult cells [74, 75]. Hence, the fetus may be intrinsically at increased risk for CMV infection upon exposure to the virus. A study of cord and adult blood-derived myeloid dendritic cells, following infection with CMV, demonstrated significantly lower levels of IL-12, IFN-*β*, and IFN-lambda1 production in neonatal cells [76]. On the other hand, another study comparing immune responses between congenitally infected infants and their mothers (who all had serological evidence of primary infection) demonstrated that neonates had significantly higher levels of IL-8, but lower levels of IF-*γ* [77]. Most of the infants in this report were asymptomatic, so the functional consequences of these differences in cytokine profiles with respect to the susceptibility, pathogenesis, and natural history of CMV infection are not clear.

The profile of chemokine and defensin production in the setting of congenital CMV infection has not been extensively studied. One study assayed cytokines and chemokines from midtrimester amniotic fluid in 8 patients giving birth to infants with congenital CMV; midtrimester sera from 12 pregnant women with primary CMV infection; and amniotic fluid and serum from uninfected pregnant controls. This analysis demonstrated that levels of chemokines CCL2, CCL4, and CXCL10 were significantly elevated in amniotic fluid from congenital CMV patients [71]. In this study, only CXCL10 was significantly elevated in the serum of CMV-infected pregnant women, compared to controls. This study did not comment on the chemokine profiles observed in the subset of women with primary CMV infections who did not go on to transmit virus to the fetus. Future studies of amniotic fluid comparing these subgroups would be of considerable interest in elucidating differences between transmitting and nontransmitting mothers. In another study of chemokine production in the CMV-infected placenta, expression of the chemokine MCP-1 was associated with fetal demise [78]. The effect was specific to CMV, insofar as other placental pathogens did not induce MCP-1 hyperexpression. These observations suggested that CMV-induced chemokine dysregulation of placental function may be an important indirect contributor to fetal disease, leading to adverse pregnancy outcome even in the absence of fetal infection *per se*. An interesting study reported by Liu and colleagues used serum proteomic analyses in an attempt to compare protein biomarkers of potential interest in infants with congenital CMV and controls. This study had subgroups of congenitally infected infants who were asymptomatic at birth and compared their proteome profiles to those of symptomatic infants with hepatitis. Thus, this study had the potential to identify candidate biomarkers associated both with a heightened risk of CMV disease or, conversely, protection against disease. Intriguingly, two protein peaks were noted that were upregulated in asymptomatic infants. These protein peaks were interpreted by these investigators, based on molecular weight, as corresponding to *β*-defensins 31 and 8 [54]. Further studies to confirm this observation and to better define the role of *β*-defensins in protection against congenital CMV infection are warranted.

## 3. Adaptive Immunity

Adaptive immunity in the context of congenital and perinatal CMV infection has clearly been more extensively evaluated than innate immunity. This reflects to a substantial extent the fact that therapeutic interventions based on adaptive immune responses, such as vaccines, are in advanced stages in clinical trials [79]. Adaptive immunity conferred by passive transfer of therapeutic anti-CMV immunoglobulin is also an area of intense clinical research activity [80, 81]. A number of studies have attempted to elucidate the role of antibody in both protection against congenital CMV transmission and, paradoxically, in promoting transmission of CMV across the syncytiotrophoblast. Evaluation of cellular immune responses has suggested for many years that there is functional impairment of aspects of cell-mediated immunity in infants with CMV infection and their mothers that may be important in transmission and disease progression. These early studies included demonstration of diminished lymphocyte-mediated cytotoxicity in infants with congenital CMV infection and their mothers compared to controls [82] and diminished CMV-specific lymphocyte blastogenesis and interferon production in congenitally infected infants and their mothers [83]. More recent definitive analyses of specific T-cell populations and of immunoglobulins in the context of congenital infection have been undertaken. These studies are reviewed in the following section.

### 3.1. CD4+ T Cells

The magnitude of the maternal CD4+ T-cell response to CMV infection appears to play an important role in predicting whether virus is transmitted to the fetus. A study of 46 pregnant and 8 nonpregnant women, seropositive for CMV and actively shedding virus in urine, examined the frequency of CMV-specific CD4+ T cells in peripheral blood lymphocytes [84]. Intracellular cytokine staining for IF-*γ* and TNF-*α* was also performed. There were no changes in the frequencies of CMV-specific CD4+ T cells in CMV-seropositive normal nonpregnant and pregnant women at any gestation, although the frequency of CMV-specific CD4+ T cells was increased in pregnant women with evidence of CMV reactivation or reinfection. There were no congenital CMV infections in these pregnancies, leading these authors to propose that the CD4+ T-cell response can contribute to protection against intrauterine transmission, particularly in the setting of exposure to either reactivated latent virus, or new strains of virus encountered in the setting of re-infection. Another prospective study examined CMV-specific lymphoproliferative response and intracellular cytokine (IFN-*γ* and IL-2) production during the first year after primary infection in 49 pregnant women and 9 nonpregnant controls. During the first month after infection, IFN-*γ* producing CD4+ and CD8+ T cells were uniformly present, whereas IL2-producing T cells were very rarely detected. Notably, a significantly delayed development of the CD4+ T-cell lymphoproliferative response was observed in infected mothers who transmitted virus to the fetus, compared with women who did not transmit [85]. 

Another study of 74 pregnant women and 29 nonpregnant controls with primary CMV infection enumerated CMV-specific CD4+ cells by cytokine flow cytometry and lymphoproliferative responses [86]. A significantly lower median stimulation index was observed in the 19 women who transmitted the virus than in the 21 women who did not. No other immunologic (IgM response, IgG antibody avidity) or virologic marker (magnitude of DNAemia) was predictive of transmission. Similar observations regarding the importance of the lymphoproliferative response to CMV have been noted by other investigators [87, 88]. These observations suggest that interventions designed to maximize the maternal CMV-specific lymphoproliferative CD4+ response may be useful in protection against congenital CMV infection. Other studies have examined the pattern of CMV-specific T-cell responses in pregnant women during the first year after acquisition of a primary infection, compared to those of pregnant women with prior preconception immunity to CMV. These analyses demonstrated that, in addition to the delayed lymphoproliferative response in CMV-transmitting mothers, there was also a significant delay in the reversion of CMV-specific effector memory T cells to the CD45RA+ phenotype [89, 90]. These investigators proposed that examination of CD45RA reexpression might be an important prognostic parameter in the setting of maternal-fetal transmission.

In addition to the importance of maternal CD4+ responses in CMV transmission, CD4+ responses in the fetus and newborn in the context of vertical transmission may also play a role in predicting the outcome of congenital infection. CD4+ responses to CMV infection in young children are of substantially diminished magnitude compared to adults. Young children have a selective and long-lived deficiency in CD4+ T cell immunity characterized by decreased IF-*γ* and IL-2 production. It was postulated that this suboptimal CD4+ response might be responsible for the prolonged shedding of CMV observed in infants following acquisition of CMV infection [91]. In this study, these young children had no symptoms of CMV disease and had presumably acquired infection from breastfeeding or attendance in group day care. 

In addition to the study of CD4+ responses in pregnant women and young children with post-natally acquired infections, the CD4+ responses of the CMV-infected fetus has been evaluated in several studies. These analyses suggest that the magnitude of the CD4+ response in the fetus may not play a significant role in protection, and in fact may correlate with the severity of CMV disease. In a study of perinatal CMV infection in a high seroprevalence population, the frequencies of CMV-specific CD4+ T cells detected by intracellular cytokine staining for IF-*γ* and TNF-*α* were higher in infants with symptomatic congenital infection than in those infants with asymptomatic perinatal infection [92]. This could, of course, simply reflect a more intense infection with higher viral load and not a deleterious effect of the cytokine response *per se*. The authors suggested that monitoring these immunological markers could be useful in predicting the prognosis of congenital CMV infection. 

Not all studies have readily demonstrated fetal/neonatal CD4+ responses in the setting of congenital infection. A study of seven patients with congenital CMV infection, six healthy infants who had acquired infection postnatally, and six CMV-seropositive adults found a striking paucity of CMV-specific IF-*γ*-producing CD4+ cells in congenitally infected infants, compared to the healthy infant and adult controls with CMV infection; however, the congenitally infected infants in this study were asymptomatic, so this study did not exclude a relationship between CD4+ response and symptomatic disease [93]. Another recent study compared T-cell responses in 24 children with congenital CMV infection (9 symptomatic), 19 children with postnatal CMV infection, and 8 adults with symptomatic primary CMV infection. Compared to adults, CMV-specific CD4+ T-cell responses in children younger than 2 years were low or undetectable, although they did appear to increase over time. No differences were noted with regard to CD8+ T-cell responses, and no differences were noted comparing symptomatic and asymptomatic children. These authors concluded that the inadequate response of CD4+ cells is a major factor responsible for lack of immune control of CMV infections in infants and young children [94]. It is of interest to reflect on these observations in light of a recent study demonstrating a striking inhibitory effect of CMV particles on CD4+ T-cell proliferation, concomitant with decreased levels of cytokines IL-4, IFN-*γ*, and TNF-*α* in cell culture [95].

An interesting subset of CD4+ cells are known as regulatory T cells (Tregs). These cells are critical to the maintenance of immune cell homeostasis by mediating a dominant negative regulation on other immune cells. These cells can be broadly classified into natural or adaptive (induced) Tregs. “Natural” Tregs are CD4+CD25+ T cells which develop in and emigrate from the thymus to maintain immune homeostasis, maintain tolerance to self-antigens, and abrogate autoimmune disease. “Adaptive” Tregs are nonregulatory CD4+ T cells which acquire CD25 expression outside of the thymus and are typically induced by inflammation and disease processes. Expansion of Tregs is important in the maintenance of normal pregnancy and contributes to the protection of the fetus from the maternal immune response [96, 97]. However, Tregs may also block beneficial immune responses by preventing development of sterilizing immunity to viruses [98, 99]. The role that Tregs play in susceptibility to or protection against fetal CMV infection has not been investigated. A recent analysis of Tregs during CMV replication in solid organ transplant recipients demonstrated that lower Tregs were observed in patients with spontaneous clearance of virus after transplantation and that the ratio of CMV-specific T cells to Tregs was highly predictive of relapse [100]. Treg-mediated suppression of anti-CMV responses was observed in a study in which Tregs were depleted from peripheral blood mononuclear cells prior to measurement of IF-*γ* production. In this study, CD8+ T cells produced more IF-*γ* in the absence of Tregs [101]. Following hematopoietic stem cell transplantation, Tregs do not appear to inhibit CMV clearance by conventional T cells [102]. The relevance of these observations to the role of Tregs in modulation of fetal and neonatal CMV infection remains to be examined.

### 3.2. CD8+ T Cells

Primary infection with CMV in immunocompetent hosts is accompanied by activation and differentiation of naïve CD8+ T cells, which become effector/memory cells capable of secreting IFN-*γ* and attacking and lysing infected target cells [103]. Studies examining the specific virally-encoded targets of CD8+ T cells have demonstrated that there is a broad and diverse repertoire of responses to many viral peptides, although reactivity against CMV proteins pp65 (ppUL83) and IE-1 appear to be of the greatest importance in control of CMV disease [104–106]. Healthy CMV-seropositive individuals devote approximately 10% of the total memory T-cell pool in the peripheral blood to CD8+ cells specific for CMV antigens [107].

CD8+ responses are readily generated following CMV infection both in young children and by the fetus *in utero*. CD8+ responses in young infants, compared to adults, are known to demonstrate focused peptide specificity and lower peptide avidity, although the peptide specificity does broaden over time [108]. The development of CD8+ responses in the setting of perinatal/congenital CMV infection has been examined by a number of groups. To attempt to elucidate the role of CD8+ responses in protection against congenital CMV infection, Pédron et al. examined 16 transmitter mothers who underwent seroconversion during the first trimester of pregnancy and their fetuses (all were positive for CMV in amniotic fluid by PCR at 17–19 weeks of gestation). Fetal and maternal blood samples were collected between the 22nd and 39th week of gestation. Activation, effector, and memory phenotypes were compared, and IF-*γ* secretion was examined. The responses were generally similar, although there was a smaller pp65-specific pool in the fetus, and fetal CTLs made less IF-*γ* in response to stimulation with a CD3 monoclonal antibody [109]. Another study in 15 CMV-infected fetuses demonstrated CD8+ responses as early as the 22nd week of gestation. Compared with controls, CMV-infected fetuses demonstrated a dramatic increase in activated and terminally differentiated CD8+ T cells [110]. However, these authors noted that cellular immunity to CMV did not appear to be fully functional, evidenced by the fact that the number of T cells capable of secreting IFN-*γ* was substantially lower after *in vitro* stimulation with CMV antigen than after exposure to stimulants such as phorbol myristate and ionomycin. 

Although these studies raise questions about the functionality of CMV-specific CD8+ responses generated *in utero*, a study lead by Marchant and colleagues examining 8 infants with congenital infection and 15 uninfected controls demonstrated the expansion and the differentiation of mature CMV-specific CD8+ cells with similar characteristics to those detected in adults. These cells demonstrated potent perforin-dependent cytolytic activity and produced abundant amounts of antiviral cytokines, particularly IF-*γ* [111]. These data support the concept of a potentially protective role for the development of fetal antiviral CD8+ T-cell responses in the control of CMV disease. These observations also suggest the provocative possibility of designing and developing prenatal vaccination strategies toward the goal of priming fetal immunity against CMV, as well as for other viral diseases [112, 113]. Further studies are clearly required to define the role of CD8+ cells—engendered both in the maternal and fetal compartments—in protection against congenital CMV infection.

### 3.3. Gammadelta T Cells

Gammadelta T cells are unconventional T cells that do not require antigen processing and major histocompatibility-complex presentation of peptide epitopes and, accordingly, can react rapidly upon activation [114]. These cells demonstrate features of both adaptive and innate cells and are described as a “bridge” between innate and adaptive immunity. A study of CMV-infected fetuses demonstrated that fetal gammadelta T cells are capable of expansion and differentiation [115]. Differentiated gammadelta T cells expressed high levels of IFN-*γ*, natural killer cell receptors, and other cytokines, and demonstrated antiviral activity. Differentiated gammadelta T cells could be detected as early as after 21 weeks of gestation. The extent to which this T-cell subset participates in antiviral defense *in utero* and in early life requires further investigation.

### 3.4. Antibody

Of all the immune effectors studied in the context of congenital and perinatal CMV transmission, perhaps the most studied are anti-CMV antibodies. This is driven in part by the intense interest in adjuvanted glycoprotein subunit vaccines engendered by recent clinical trials designed to elicit a protective antibody response against CMV gB. In both young women of childbearing age [116] as well as in solid organ transplant recipients [117], a CMV vaccine based on purified subunit gB demonstrated some degree of protection against acquisition of CMV infection. The fact that the gB vaccine was also capable of boosting antibody titers when administered to women who already had CMV antibody from previous infection suggests that a vaccine strategy of immunizing seropositives might be able to prevent reinfection with and subsequent transmission of new CMV strains in women with prior immunity [118, 119]. These successes notwithstanding, the role that anti-CMV antibody response plays in protection of the fetus remains incompletely understood. Notably, there is not known to be an increased incidence of congenital CMV infection in the setting of humoral immunodeficiencies, suggesting that antibody is not absolutely required for protection. However, a case of congenital CMV infection was recently reported in a woman receiving the anti-B cell monoclonal antibody, rituximab [120], suggesting that the inability to sustain a humoral response to CMV may confer an increased risk of transmission in some patients. Rituximab has also been associated with serious CMV disease in a patient on maintenance therapy [121]. 

Although antibody plays an important role in protection against CMV infection and disease, the level of protection is clearly incomplete. Notably, CMV can readily infect the newborn infant, via ingestion of breast milk, even in the setting of passively acquired maternal antibody [17, 107]. Moreover, CMV reinfection of the pregnant woman with subsequent transmission to the fetus, as noted earlier, can occur even in the setting of preexisting maternal immunity [7, 122, 123]. These shortcomings aside, there is an emerging role for IgG as an immunotherapy for prevention and treatment of congenital CMV. A study of administration of CMV-specific hyperimmune globulin to pregnant women appeared to significantly lower the risk of congenital CMV infection and disease, although given the uncontrolled nature of the study, conclusions about the mechanism of protection could not be definitively drawn [124, 125]. The extent to which antibody therapy reverses established CMV disease in the infected fetus or prevents sequelae is uncertain, although some studies to date are very encouraging, suggesting both short-term [126] and long-term [127] benefits. The beneficial effect of immune globulin is proposed to be mediated by virus neutralization in the CMV-infected fetus, although it is possible that the benefit of IgG may be via another mechanism. The major target of the neutralizing antibody response in CMV hyperimmune globulin is directed at proteins in the gH/gL/UL128/UL130/UL131 complex [128], and if ongoing trials confirm a protective/therapeutic effect of hyperimmune globulin administration to pregnant women at high risk of CMV transmission to their fetuses [129], antibodies to this complex may emerge as an important serological correlate of protection. 

In addition to providing a protective/therapeutic effect for the CMV-infected fetus, antibody appears to exert protection at the level of the placenta. In an ultrasonographic assessment of placental thickness, women with primary CMV infection who had a fetus or newborn with CMV disease had placentas that were significantly thicker than those of women with primary CMV infection who did not have a diseased fetus or newborn; moreover, receipt of hyperimmune globulin was associated with statistically significant reductions in placental thickness [130]. Immunohistochemical analyses also supported a benefit of IgG on placental health [131]. On the other hand, CMV antibody may, paradoxically, promote transmission of virus to the fetus, via the expression of the neonatal Fc receptor on syncytiotrophoblast. It has been shown that antibody-virus complexes can translocate the syncytial barrier via this receptor, allowing entry of virus into the fetal circulation [132]. In this model, virus transmission can be interrupted if the antibody is of sufficiently high neutralizing capacity and avidity. Viral transmission from mother to fetus may be increased if the maternal antibody response is of low avidity or of poor neutralizing activity.

There is relatively little information about the ability of the infected fetus to mount an independent antibody response to CMV infection. The infected fetus generates IgM antibodies to CMV but the antiviral activity, if any, of such antibodies has not been evaluated. CMV IgM antibodies can be measured in the newborn as an adjunct to other diagnostic studies [133] but it is unknown if they play any role in disease control. 

## 4. Summary

In summary, there is limited information about the precise protective correlates of anti-CMV immunity in the setting of congenital and perinatal infection, although the increasing availability of cohorts of congenitally infected infants has enabled some research into the fetal and neonatal immune response in recent years. Protection of the fetus cannot be assured in the setting of maternal immunity, since reinfection with a novel strain of CMV can occur, with transmission to the fetus. Viral strain variation may contribute to reinfection, and low maternal IgG avidity may paradoxically promote transmission of virus across the syncytiotrophoblast, via the neonatal Fc receptor. Other immune mechanisms operating at the level of the placenta, particularly innate immunity, may play a more important role than antibody in limiting infection of the fetus. The physiological state of pregnancy may impair some aspects of the immune response to CMV, predisposing to placental and fetal infection. Tables 1 and 2 provide a summary of available information regarding the potential protective and predisposing parameters of innate and adaptive immune responses associated with CMV transmission. Importantly, the fetus can mount an immune response to CMV, although the role that this response plays in limiting the extent of CMV-associated disease or sequelae remains uncertain and requires further investigation. It is conceivable that novel vaccines and immunotherapies could exploit aspects of innate and adaptive immunity known to correlate with protection. These should be considered in ongoing vaccine design. It is also important to keep in perspective that not all congenital infections result in disease and/or sequelae. Prospective natural history studies that define the immune correlates associated with protection against transmission and/or protection of the newborn from progression to CMV disease are needed. Since most congenitally infected infants are asymptomatic and have a good prognosis for normal neurodevelopmental outcome, comparing the immunological profile of asymptomatic infants to those with disease and/or sequelae is a high-priority area for future research. These observations could in turn provide opportunities for the design and development of effective interventions to help address this unsolved public health problem.

## Figures and Tables

**Table 1:** Summary of innate immune responses and their proposed role in control of or susceptibility to congenital CMV infection.

Innate immunity and susceptibility/protection in congenital CMV infection		
Immune effector	Maternal/placental/fetal compartment	Proposed effect on CMV transmission/disease
NK cells-CD56^bright^	Maternal (pregnancy) Uterine NK cells	(i) Decreased cytolytic potential (ii) Increased risk of CMV transmission?

NK cells-NKG2C+	Fetal compartment	(i) Expansion of this NK subset in congenital and perinatal CMV (ii) Correlation with symptomatic CMV disease?

Phagocytic cells	Placental compartment	(i) Neutrophils: possible role in defense (ii) Macrophage: potentiates spread to syncytiotrophoblasts?

Toll-like receptors	Maternal compartment Placental compartment	(i) TLR2 polymorphism; ↓ signaling to CMV glycoproteins; ↑ risk of CMV disease in transplant patients; increased transmission risk? (ii) TLR3 polymorphism; decreased signaling to CMV antigens (iii) TLR7 polymorphism: decreased antibody response to glycoprotein B?

Cytokines Chemokines Defensins	Neonatal compartment Maternal compartment Placental compartment Placental-fetal interface	(i) ↑ IL-8 ↓ IF-*γ* may correlate with increased transmission risk (ii) Increased maternal CCL-10 correlates with transmission (iii) Increased placental MCP-1 expression correlates with fetal demise (iv) Physiological increase in uterine IL-10 in pregnancy: increased risk of reactivation/transmission? (v) Beta-defensins 8 and 31 proposed to be upregulated in amniotic fluid of asymptomatically congenitally infected infants

**Table 2:** Summary of adaptive immune responses and their proposed role in control of or susceptibility to congenital CMV infection.

Adaptive immunity and susceptibility/protection in congenital CMV infection		
Immune effector	Maternal/placental/fetal compartment	Proposed effect on CMV transmission/disease
CD4+ T cells	Maternal compartment Fetal/neonatal compartment	(i) Delayed development of CD4+ T-cell lymphoproliferative response correlates with maternal-fetal transmission (ii) Defective CD4+ immunity; diminished IF-*γ* and IL-2 production in fetal and early childhood infection (iii) Defective fetal CD4+ response may contribute to congenital CMV infection and disease

T-regs	Maternal compartment	(i) Treg expansion: normal response to pregnancy (ii) ↓ Tregs: correlates with protection against CMV disease in transplant recipients (iii) Relevance to congenital CMV unknown

CD8+ T cells	Maternal compartment Fetal/neonatal compartment	(i) CD8+ response to infection appears unaltered in pregnancy (ii) Fetal CD8+ response to CMV antigens noted as early as week 22 gestation (iii) Exhibit cytolytic properties and elucidate IF-*γ* (iv) Some studies raise questions about functionality?

Gammadelta T cells	Neonatal compartment	(i) Fetal gammadelta T cells differentiate and expand in setting of congenital CMV infection (ii) Produce IF-*γ* and other cytokines (iii) Role in protection of fetus, control of virus not clear

Antibody	Maternal compartment Placental compartment Fetal compartment	(i) Variability in maternal antibody response based on viral strain variation, possibly TLR polymorphisms (ii) Expression of neonatal Fc receptor may paradoxically promote transcytosis of CMV particles across syncytiotrophoblast by low-avidity antibody (iii) High avidity antibody may neutralize CMV at placental interface (iv) Transplacental transfer of therapeutic neutralizing antibody may improve outcome of infected fetus
